# Supplementary material for: In Vivo Metabolic Response upon Exposure to Gold Nanorod Core/Silver Shell Nanostructures: Modulation of Inflammation and Upregulation of Dopamine
Source: Int J Mol Sci. 2020 Jan 8;21(2):384. doi: 10.3390/ijms21020384 (PMC7014015; doi:10.3390/ijms21020384)
Supplement: Supplementary file 1 [file ijms-21-00384-s001.pdf]

Supporting Information

Metabolism response to gold nanorod core/silver shell nanostructures: modulation of inflammation and upregulation dopamine

Haiyun Li [1,2]#, Tao Wen [3]#, Tao Wang [3], Yinglu Ji [1], Yaoyi Shen [3], Jiaqi Chen [1,2], Haiyan Xu [3*], and Xiaochun Wu [1,2*]

[1] CAS Key Laboratory of Standardization and Measurement for Nanotechnology & CAS Center for Excellence in Nanoscience, National Center for Nanoscience and Technology, Beijing 100190, China

[2] University of Chinese Academy of Science, Beijing 100049, China

[3] Institute of Basic Medical Sciences, Chinese Academy of Medical Sciences & Peking Union Medical College, Beijing 100730, China;

† Equal contribution

* Correspondence: wuxc@nanoctr.cn; xuhy@pumc.edu.cn;

# These authors contributed equally to this work.

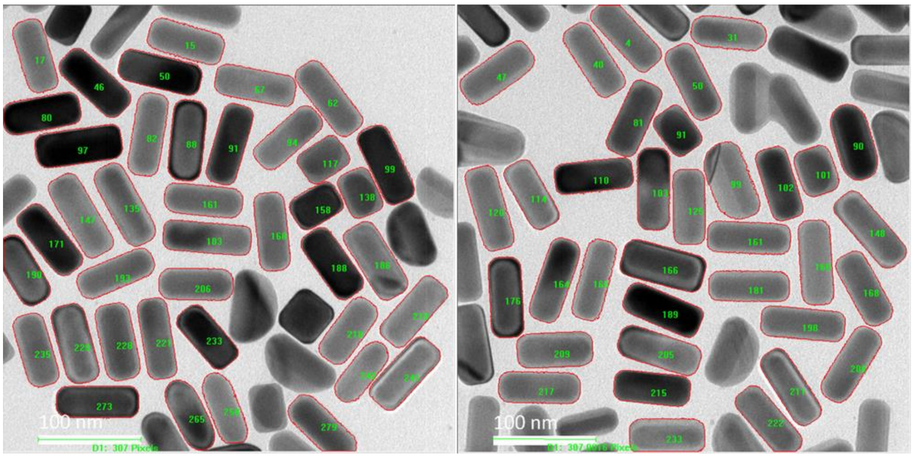

Figure S1. TEM images of Au@Ag NRs used for calculation of length and width. The mean length and width of NRs are 78.0 ± 4.1 nm, 32.4 ± 1.7 nm, respectively. The mean aspect ratio was 2.41 ± 0.16. Data were presented as mean ± SD (n=64).

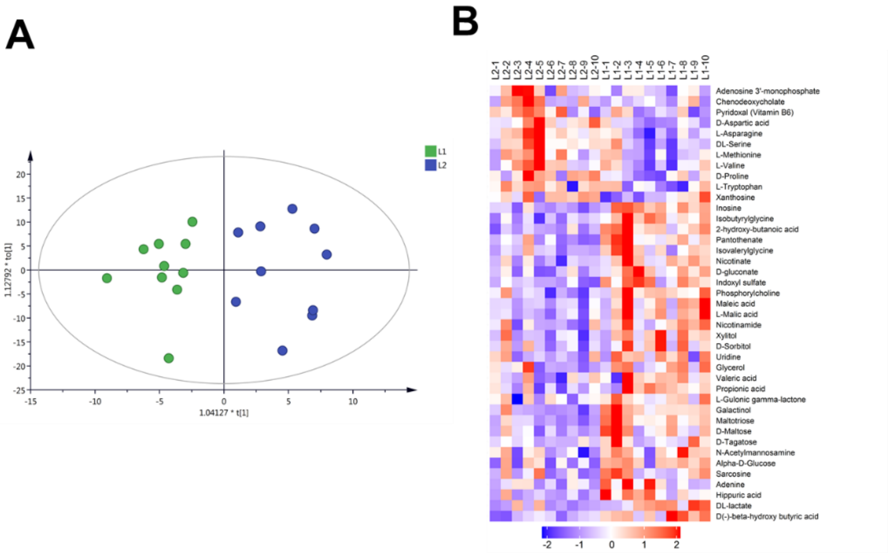

Figure S2. Effects of Au@Ag NRs on metabolomics in mouse liver. (A) Metabolic cluster analysis using an OPLS-DA scores plot. L1-N is the control group and L2-N is the mice received multiple administration of Au@Ag NRs. (N=1-10) (B) Heat maps of differential metabolites. All the data were obtained under negative mode

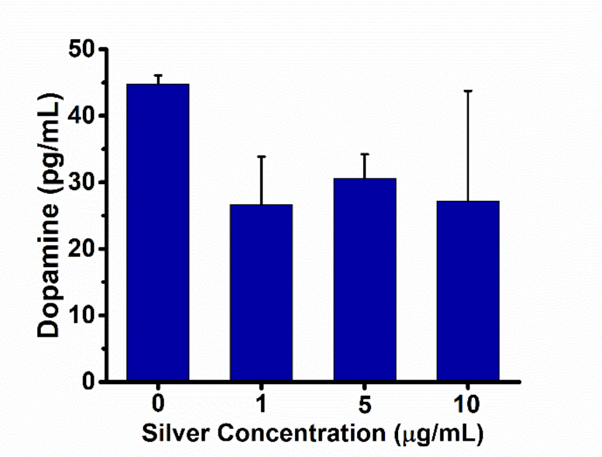

Figure S3. Effects of Au@Ag NRs on secretion of dopamine in Treg cells.

Table S1. variant metabolites under positive mode

description	VIP	Fold change	p-value
Isobutyrylglycine	1.3322	0.60791217	2.5692E-05
D-Pipecolinic acid	3.53555	1.66548969	0.00017631
Xanthine	3.20653	0.89372768	0.00040351
Cyclohexylamine	7.00695	0.66302259	0.0004145
Maltotriose	4.17924	0.67505953	0.0004685
Adenine	2.30405	0.84082096	0.00053748
L-Pipecolic acid	3.83812	1.66483164	0.00054871

Cellobiose	3.6097	0.73271788	0.00078811
Pantothenate	2.7699	0.81180502	0.00094477
Urea	1.21345	0.80520621	0.00106664
1-Stearoyl-sn-glycerol 3-phosphocholine	7.59854	0.72367516	0.0022091
Anthranilic acid (Vitamin L1)	3.23127	0.65551114	0.00318306
D-Proline	4.06984	1.61789939	0.00329891
Inosine	4.63986	0.69643213	0.00350906
L-Asparagine	2.12866	1.21542424	0.00380376
1-Stearoyl-2-oleoyl-sn-glycerol 3-phosphocholine (SOPC)	3.54098	2.82892395	0.0039038
Dopamine	1.19994	1.18084728	0.00396378
trans-2-Hydroxycinnamic acid	1.68733	1.18850602	0.00488449
S-Methyl-5'-thioadenosine	4.4996	0.73810755	0.004953
L-Serine	1.37818	1.17815014	0.00540942
1-Oleoyl-sn-glycero-3-phosphocholine	7.27013	0.79983609	0.00569728
Hypoxanthine	2.56096	0.93916518	0.00598881
L-Aspartate	1.73308	1.21460591	0.00656762
L-Tyrosine	2.80009	1.18045181	0.00661534
Ergothioneine	2.00823	0.87294863	0.01083809
1-Palmitoyl-sn-glycero-3-phosphocholine	6.09609	0.74498179	0.01194912
Thioetheramide-PC	5.29022	1.45773406	0.01291098
3.alpha.-Mannobiose	1.88126	0.77236606	0.01712782
Isomaltose	9.93102	0.77518386	0.017502
PC(16:0/16:0)	4.06947	1.96678631	0.01953661
Creatinine	1.15527	0.8217466	0.01965534
Choline	1.0697	0.85027673	0.02051861
Pyridoxine	2.93676	0.65860961	0.02167676
Glycerophosphocholine	6.89478	0.83780361	0.02285505
L-Methionine	3.08467	1.20469105	0.03758204
Nicotinamide	6.21993	0.903752	0.0432002
L-Norleucine	1.12535	1.25213158	0.04327327
Cholic acid	5.21803	1.30938596	0.04986696
Stachyose	1.14017	0.72687697	0.05828351
1,2-dioleoyl-sn-glycero-3-phosphatidylcholine	2.33406	1.2493403	0.06746331
Oxypurinol	1.20717	1.12287762	0.07102895
Xanthosine	1.54988	1.12057893	0.07541243
1-Myristoyl-sn-glycero-3-phosphocholine	1.90661	0.75621031	0.07697028
L-Phenylalanine	5.14322	1.13967865	0.07852509
Uridine	3.25877	0.87253389	0.08081724
1-Methylnicotinamide	1.37866	1.3355248	0.09180785
Uracil	1.35565	0.89908537	0.09610525

Table S2. variant metabolites under negative mode

adduct	description	VIP	Fold change	p-value
(M-H2O-H)-	Inosine	4.23277	0.6951192	2.3713E-05
(M-H)-	2-hydroxy-butanoic acid	2.48478	0.61857415	7.8275E-05
(M-H)-	Isobutyrylglycine	2.50611	0.6082409	0.00013319
(M-H)-	D(-)-beta-hydroxy butyric acid	1.85184	0.62636174	0.00020649
(M+CH3COO)-	Maltotriose	2.80205	0.59856512	0.00022519
(M-H)-	Indoxyl sulfate	4.99509	0.51079081	0.00034536
(2M-H)-	Galactinol	1.2864	0.5440873	0.0005412
(M-H)-	D-gluconate	2.82247	0.70678267	0.00176661
(M-H)-	Pantothenate	3.34705	0.81701895	0.00206136
(M-H)-	L-Malic acid	3.14375	0.85202397	0.00226071
(M-H)-	L-Asparagine	2.38519	1.20379884	0.00319813
(M-H)-	Nicotinate	1.06332	0.75123281	0.00327574
(M+CH3COO)-	Phosphorylcholine	1.79248	0.71367261	0.00376836
(M-H)-	D-Aspartic acid	2.56867	1.24641671	0.00426662
(M-H)-	Hippuric acid	1.33922	0.45142596	0.00431605
(M-H)-	Maleic acid	1.02241	0.85392362	0.00523615
(M-H)-	DL-lactate	3.10708	0.6335213	0.00693748
(M-H)-	D-Maltose	7.07192	0.84128333	0.00921171
(M-H)-	Isovalerylglycine	1.89819	0.80192767	0.01304613
(M-H)-	Alpha-D-Glucose	18.5136	0.72509872	0.01406511
(M-H)-	DL-Serine	1.76545	1.16721891	0.0147534
(M-H)-	D-Proline	2.00206	1.28449458	0.01734674
(M+CH3COO)-	Xylitol	1.764	0.74860469	0.01779102
(M-H)-	Propionic acid	1.16006	0.70110257	0.01808621
(M-H)-	Nicotinamide	1.40073	0.89608788	0.0306026
(M-H)-	N-Acetylmannosamine	1.25266	0.88179801	0.0425275
(M-H)-	L-Methionine	2.3993	1.19515828	0.05146904
(M-H)-	Xanthosine	1.85418	1.13462267	0.05953812
(M-H)-	Adenine	1.09896	0.83945206	0.06227195
(M-H)-	L-Valine	2.31342	1.14956767	0.06493521
(M-H)-	Pyridoxal (Vitamin B6)	1.21098	1.34030817	0.06551082
(M-H)-	D-Sorbitol	1.89474	0.84348332	0.06804239
(M+CH3COO)-	Glycerol	1.83996	0.88956665	0.07343579
(M-H)-	Adenosine 3'-monophosphate	1.01864	1.18810691	0.07857589
(M-H)-	Sarcosine	1.28121	0.8903312	0.08464105
(M-H2O-H)-	D-Tagatose	1.06183	0.57800184	0.08486897
(M-H)-	Uridine	9.06442	0.8532209	0.08701019
(M-H)-	L-Gulonic gamma-lactone	1.48168	0.89742768	0.08808941

(M-H)-	L-Tryptophan	1.75658	1.40043264	0.09441359
(M-H)-	Valeric acid	1.59113	0.76679659	0.09554984
(M+CH3COO)-	Chenodeoxycholate	2.19638	1.6639374	0.09744304
